# Application of Finite Element Analysis Combined With Virtual Computer in Preoperative Planning of Distal Femoral Fracture

**DOI:** 10.3389/fsurg.2022.803541

**Published:** 2022-02-22

**Authors:** Yuanming He, Yang Liu, Bo Yin, Dong Wang, Hanzhou Wang, Peifeng Yao, Junlin Zhou

**Affiliations:** Department of Orthopedic Surgery, Beijing Chaoyang Hospital, Capital Medical University, Beijing, China

**Keywords:** computer-assisted preoperative planning, distal femoral fractures, finite element analysis, 3D model, plate length, screw position

## Abstract

**Background:**

Distal femoral fractures are increasing with an aging population. The computer-assisted preoperative planning has great potential, but there are no preoperative plans to determine appropriate fixation methods for distal femoral fractures on an individual basis. The aims of this study are: (1) to describe the technique of finite element analysis combined with computer-assisted preoperative planning to determine a fixation method for distal femoral fractures and (2) to evaluate the intra-operative realization of this technology and the clinical outcomes based on it for distal femoral fractures.

**Materials and Methods:**

Between January 2017 and January 2020, 31 patients with distal femoral fractures treated by open reduction and internal fixation were included and randomly divided into two groups based on preoperative planning methods: conventional group (*n* = 15) and computer-assisted group (*n* = 16). Firstly, how to determine the most appropriate plate and screw length and placement in the preoperative planning of distal femoral fractures was described. The time taken for preoperative planning for different fracture types in the computer-assisted group was then analyzed. Finally, intraoperative and postoperative parameters were compared between the conventional and computer-assisted groups, assessing operative time, intraoperative blood loss, number of intraoperative fluoroscopies, days of hospital stay, Visual Analog Scale for Pain Score (VAS), and Knee Society Score (KSS).

**Results:**

Mean total planning time for 33-A, 33-B, and 33-C fractures in computer-assisted group were 194.8 ± 6.49, 163.71 ± 9.22, and 237 ± 5.33 min, respectively. Compared with the conventional group, the patients in the computer-assisted group had less blood loss, fewer fluoroscopic images, and shorter operation time (*p* < 0.05). However, there was no significant difference in the hospitalization days, KSS score and VAS score between the two groups (*p* > 0.05).

**Conclusions:**

The results of this study show that finite element combined with computer-assisted preoperative planning can effectively help surgeons to make accurate and clinically relevant preoperative planning for distal femoral fractures, especially in the selection of appropriate plate length and screw positioning.

## Introduction

Distal femoral fractures account for 4–6% of all femoral fractures and approximately 1% of all fractures, and its incidence is gradually increasing as the population ages and periprosthetic fractures intensify (1). Distal femoral fractures represent one of the largest challenges in the surgical treatment of fractures, with a reported mortality rate of 18 to 30% within 1 year for elderly people with distal femoral fractures, similar to that of hip fractures (2, 3). Surgical treatment of distal femoral fractures includes open reduction and internal fixation, joint replacement, etc. (4, 5). All these methods have the characteristics of long operation time and large blood loss, which can cause complications such as fracture, non-union and traumatic arthritis (6, 7). Therefore, appropriate preoperative planning is necessary for the treatment of distal femoral fractures. An effective preoperative plan needs to be made after an adequate assessment of the patient's physical condition and fracture status by clinicians. The use of preoperative planning can help physicians to select the appropriate treatment, improve the efficacy of the treatment, and reduce complications.

In the past, traditional preoperative planning required surgeons to view the X-ray and computed tomography (CT) images of patients to analyze their fracture conditions and select treatments (8). This image-based viewing method is intuitive, fast, and can analyze the situation and treatment of simple fractures. However, for complex fractures, such as high-energy fractures containing a large number of bone fragments, this simple method often fails to enable surgeons to have a clear understanding of the fracture situation. The type of internal fixation for fractures can only be manually selected after intra-operative exposure of the fractured end. With advances in medical imaging, 3D reconstruction of CT images enables surgeons to observe complex fractures from multiple angles (9). Preoperative planning plays an important role. In addition to helping surgeons understand fracture morphology, an efficient preoperative plan can also help surgeons to select reduction methods and the type of internal fixation models. 3D printing technology based on CT scans has become a preoperative planning tool used by many surgeons. Surgeons can directly observe and touch the solid model, which is printed after the CT images are processed by the computer (10, 11). Through the reduction of the solid model and the selection of internal fixation, the surgeon can improve the treatment effect through virtual surgery. However, the printing of the solid model takes a long time and involves additional costs. These disadvantages limit the use of 3D printing technique in preoperative planning. Computer-assisted virtual preoperative planning addresses these disadvantages by allowing the surgeon to simulate fracture reduction, measure anatomical parameters, and select internal fixation dimensions in specific software (12, 13). Computer-assisted virtual preoperative planning also has some shortcomings. The currently designed computer-assisted virtual preoperative planning still cannot analyze whether the fixation method selected in the preoperative planning is suitable for fracture healing. In other words, the surgeon cannot determine the biomechanical strength of the chosen internal fixation method.

Finite element analysis is a computer-based method of approximating values by dividing the bone into smaller elements and calculating the stresses and strains in each element. Since the 1970s, finite element analysis has been developed to study bone and joint diseases, bone mass detection, and implant design. Finite element analysis can simulate the mechanical response of bones under load to simulate physiological and traumatic loading as well as orthopedic reconstruction behavior (14). Non-invasive assessment of fracture end mechanics under different load strengths is important in clinical practice (15). Finite element analysis can be used to calculate the biomechanical strength of different fixation methods and the fracture end mechanics of patients with different physical conditions (16).

In this paper, we present a method for preoperative planning of distal femoral fractures by combining finite element analysis methods with computer simulation. It is hypothesized that this method will facilitate the orthopedic surgeon in selecting the appropriate treatment plan for different fracture conditions of the distal femur and thus improve the outcome. The aims of this study are: (1) to describe how finite element analysis methods can be used in preoperative planning of distal femoral fractures in conjunction with preoperative planning software. (2) To compare the preoperative use and results of the new and traditional methods.

## Methods

### Patients and Methods

The study was conducted in accordance with the ethical standards set out in the Declaration of Helsinki and its subsequent amendments. The design of the study was approved by the Ethics Committee of the Department of Orthopedics, Beijing Chaoyang Hospital, Capital Medical University (310, 2017).

In this prospective study, 31 patients with distal femoral fractures requiring surgical treatment were included between January 2017 and January 2020. Patient selection was based on inclusion-exclusion criteria (Table 1). All the patients were randomly divided into two groups according to the order of admission of odd and even numbers: conventional group and computer-assisted group. Sixteen patients with distal femoral fractures were selected as the computer-assisted group to make preoperative planning by combining finite element analysis with computer simulation, and 15 patients with distal femoral fractures were selected as the conventional group to make preoperative planning by routine viewing of X-ray and CT images. Data from both groups were collected using a medical record system and picture archiving communication system (PACS) to determine patient demographics, comorbidities, and preoperative clinical data (Table 2).

**Table 1:** Inclusion-exclusion criteria.

**Item**	**Description**
Inclusion criteria	1. Closed fracture
	2. Fresh fracture
	3. Treatment with plate and screw fixation.
	3. No neurological injury
	4. Informed consent form signed preoperatively
Exclusion criteria	1. Open fractures
	2. Non-displaced fractures
	3. Multiple trauma
	4. Unable to follow postoperative recommendations
	5. Pathological fractures

**Table 2:** Patient demographics.

	**Conventional group (*N* = 15)**	**Virtual surgical group (*N* = 16)**	***P*-Value**
Age^*^	65.6 (33–90)	69.7 (49–90)	0.332
Gender^§^			0.829
Male	4 (0.27)	5 (0.31)	
Female	11 (0.73)	11 (0.69)	
AO/OTA classification^§^			0.978
33-A	5 (0.33)	5 (0.31)	
33-B	6 (0.40)	7 (0.44)	
33-C	4 (0.27)	4 (0.25)	
Injured side^§^			0.870
Left	7(0.47)	7(0.44)	
Right	8(0.53)	9(0.56)	
Diabetes^§^	2(0.13)	3(0.19)	0.682
Smoking status^§^	2(0.13)	4(0.25)	0.411

* *The values are given as the mean, with the range in parentheses*.

§ *The values are given as the number, with the percentage in parentheses*.

### Preoperative Evaluation and Planning

In the computer-assisted group, the CT image data of patients were subjected to three-dimensional reconstruction, fragment division, and virtual reduction. Then the steel plate and screw were assembled on the simulated reduced bone. Finally, the finite element method was used to conduct biomechanical analysis on different fixation methods, and the stress and strain under different fixation schemes were compared to select the length and position of the steel plate and screw that were most suitable for patients. The specific steps are as follows.

**Figure 1:**
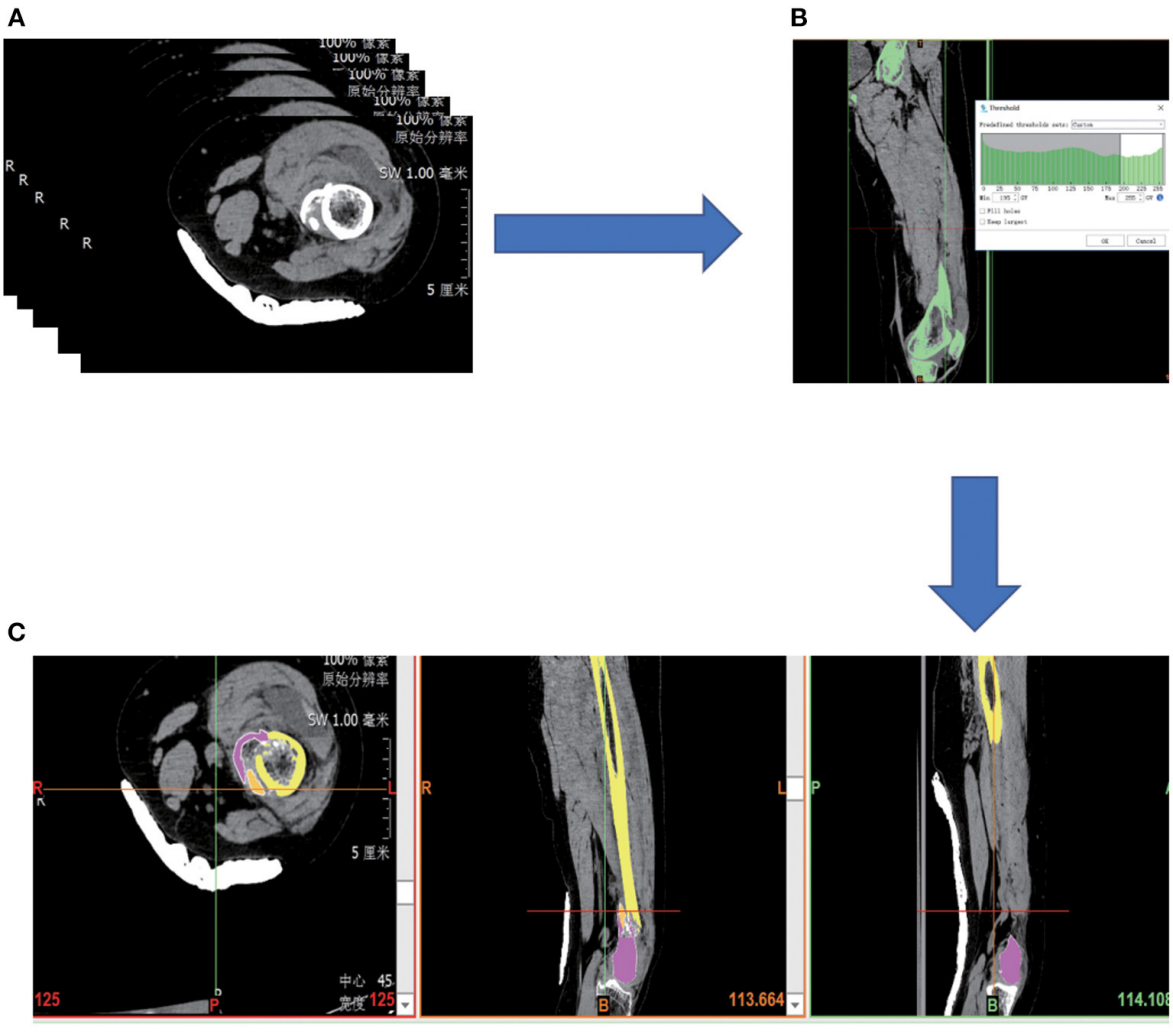
**(A)** Collect patient image data in PACS system. **(B)** Select bones by thresholding. **(C)** Fracture fragments were divided in different colors.

The CT images were imported into Mimics Medical 21.0 (Materialize, Leuven, Belgium) to calculate a volumetric dataset. The 3D model of the femur was constructed by thresholding, region growing, and editing masks according to the differences in the gray values of the different tissues, and the different fracture fragments were labeled with different colors (Figure 1). The fracture fragments were then simulated for reduction. The data set was then saved and exported in STL format (Stereolithography, Standard Template Library).

**Figure 2:**
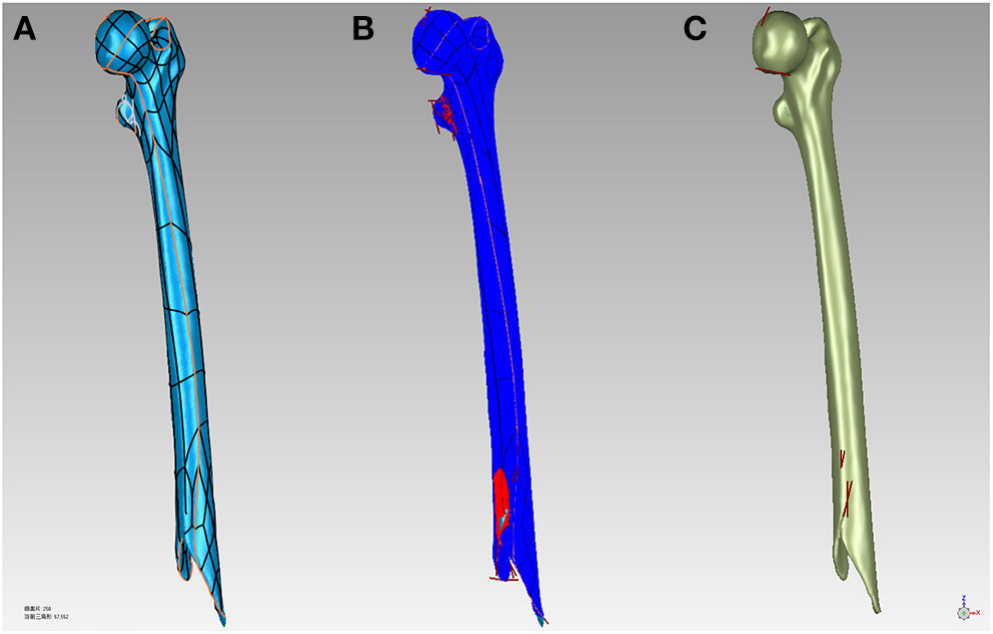
**(A)** Build surface patches to bone surface. **(B)** Repair the model defects. **(C)** Fit to NURBS surfaces to the bone.

Using Geomagic Studio 2017 (Geomagic control; 3D Systems) to simplify, smooth, and further optimize the STL model, and then create boundary constraints. Once the boundary constraints are created, the patches are constructed by data partitioning based on the constraints. Then, the grid is constructed on the basis of the surface patches. Finally, these features extracted and edited in the polygon stage are automatically fitted to a NURBS (Non-Uniform Rational B-Splines) surface (Figure 2).

**Figure 3:**
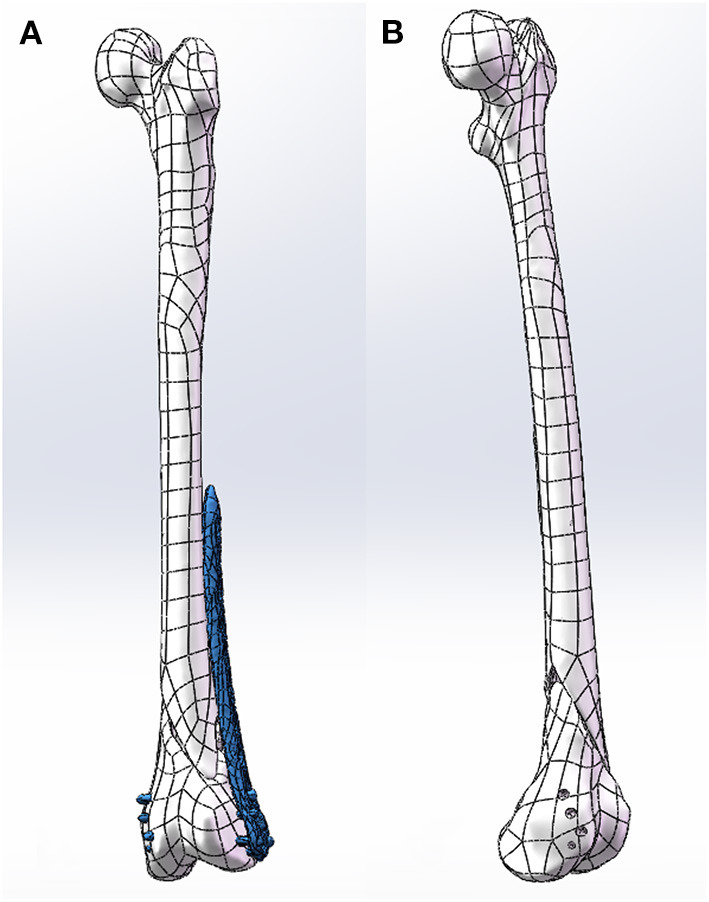
**(A)** Clinically place plates and screws on the bone. **(B)** Model the screw holes in the bone.

After the construction of the NURBS surface, the NURBS surface was generated as an SLDPRT part using SolidWorks (SolidWorks Corp, MA, USA) and assembled with the internal fixation. Internal fixation was selected from the pre-scan internal fixation database, and virtual placement of the plates followed clinical guidelines. The length of the screw is selected after measuring the width of the bone. The screw of an appropriate length was inserted into the screw hole of the plate and the hole of the nail in the bone and was then modeled to produce finite element analysis results (Figure 3).

**Figure 4:**
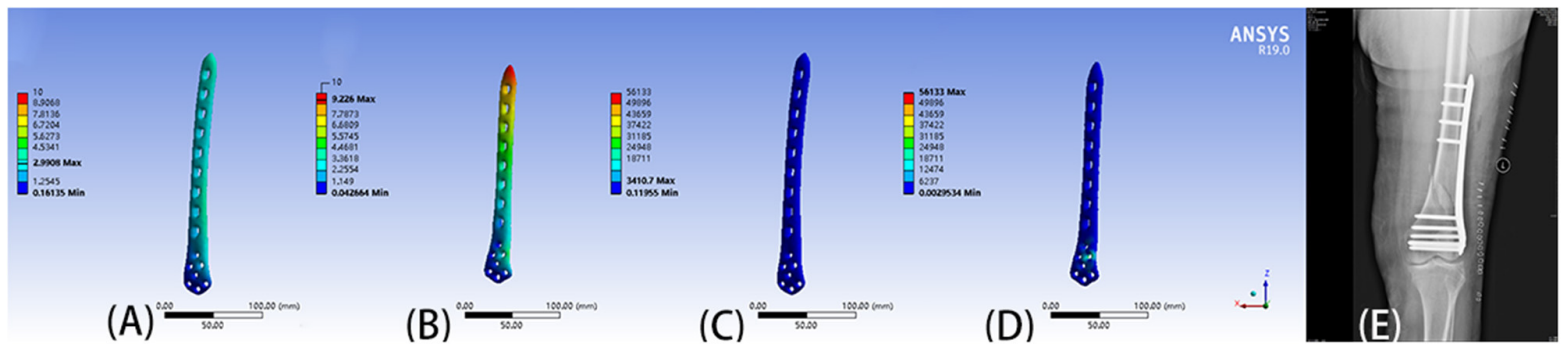
**(A)** Displacement of 9-hole steel plate (mm). **(B)** Displacement of 8-hole steel plate (mm). **(C)** The von Mises stress (Mpa) of 9-hole steel plate. **(D)** The von Mises stress (Mpa) of 8-hole steel plate. **(E)** The postoperative X-ray film showed that the operation method was similar to the preoperative plan.

Ansys 19.0 (Ansys, Inc., Canonsburg, PA, USA) was used to carry out the finite element analysis on the biomechanics of the fixation method. Firstly, the properties of materials were assigned by using the accepted gray value calculation formula and the data provided by the manufacturer such as Nobakhti et al. (17): Density = 1,017 × Grayvalue−13.4 (g/cm^3^), E-Modulus = 5,925 × Density−388.8 (MPa), the bony properties were assigned an elastic modulus Then the fixation and bone were divided into tetrahedral meshes. Finally, the weight of an adult standing upright on one leg was simulated as a load, and finite element analysis was carried out. Different fixation methods lead to different mechanical environments at the fracture end, and the appropriate method was selected after comparison. This step is particularly important when the fixation method in the preoperative plan is unclear, as it can provide a theoretical basis for clinicians to choose an internal fixation protocol (Figure 4).

In the conventional group, the surgeon understood fracture morphology by reading X-ray and CT images of patients, and made a preoperative planning decision on the length and position of the steel plate and screw according to their own experience.

### Surgical Method

The patient was under general or intraspinal anesthesia in the supine position and a tourniquet was applied to the upper third of the thigh on the affected side. The skin and subcutaneous tissue were opened layer by layer until a clear fractured end was exposed. The fracture was then reduced and temporarily fixed with Kirschner wires. For more severe 33-C fractures, an additional auxiliary incision was made as needed to expose the medial condyle and the fractured end of the medial femoral fracture and restore. C-arm fluoroscopy showed that the reduction of the fracture was satisfactory, and internal fixation of the fracture was performed.

In the computer-assisted group, fracture fixation was performed according to the optimal fixation scheme of finite element analysis. In the conventional group, the appropriate plate length and position was selected according to the treatment principle and surgeon experience, and the appropriate screw length was selected by manual measurement.

All surgeries were performed by a senior surgeon (J. Z.) with 20 years of clinical experience in the treatment of distal femoral fractures. After surgery, the affected limb was raised to relieve the soft tissue swelling around the wound. The drainage tube was removed the next day after the operation, and the flexion-extension movements of the knee joint were performed. Partial weight-bearing was permitted 8 weeks after surgery. Only when the imaging results were satisfactory the patient could load the limb completely.

Operation time, intraoperative blood loss, fluoroscopy times, and hospitalization days were recorded for the two groups. Follow-up was performed at least 12 months later to evaluate the surgical effect. To quantify the surgical effect, Visual analog scale (VAS), knee joint association scale (KSS), and knee range of motion were used to evaluate the rehabilitation of patients.

### Statistical Analysis

All statistical analyses were performed using SPSS 25.0 (StataCorp, University City, USA). Data are shown as range, mean and standard deviation (SD). The Shapiro-Wilk test was used to test the normality of the data. Fisher's exact test to compare gender, diabetes, smoking, site of injury. Mann-Whitney's U-test or the Kruskal–Wallis method test the age, fracture staging and Clinical outcomes. Pearson Chi-square for AO/OTA classification. Using Kruskal-W Allis test, the time required for computer-assisted group in different stages was also compared. Significance was defined as *p* < 0.05.

## Results

Preoperative planning was completed in all of the computer-assisted groups using the proposed method. Table 3 shows the time taken for each stage of computer-assisted preoperative planning. The average time taken was 194.29 min (range 145 to 244 min). The time spent in each computer planning phase was the segmentation of the fracture fragment which completed in the software in an average time of 38.12 min, the repositioning of the fracture block in 18.89 min, the reverse modeling in 16.47 min, the virtual placement of the internal fixation in 56.76 min and the finite element analysis in 64.06 min.

**Table 3:** Time spent in stages of computer-assisted preoperative planning for different subtypes of distal femoral fractures.

	**AO/OTA 33-A**	**AO/OTA 33-B**	**AO/OTA33-C**	**Total**
Fracture fragments segmentation	39.4 ± 3.14	29.57 ± 4.50	48.8 ± 2.99	38.12 ± 8.83
Simulated reduction	19.8 ± 2.99	12.29 ± 2.71	27.2 ± 3.31	18.89 ± 6.89
Reverse modeling	15.6 ± 1.62	10.42 ± 1.40	25.8 ± 2.48	16.47 ± 6.65
Simulated implantation	57.8 ± 5.08	50.29 ± 4.53	64.8 ± 2.64	56.76 ± 7.39
FEA analyze	64 ± 2.83	59.57 ± 2.97	70.4 ± 1.85	64.06 ± 5.21
Total	194.8 ± 6.49	163.71 ± 9.22	237 ± 5.33	194.29 ± 31.81

*The values are given as the mean and the standard deviation*.

**Figure 5:**
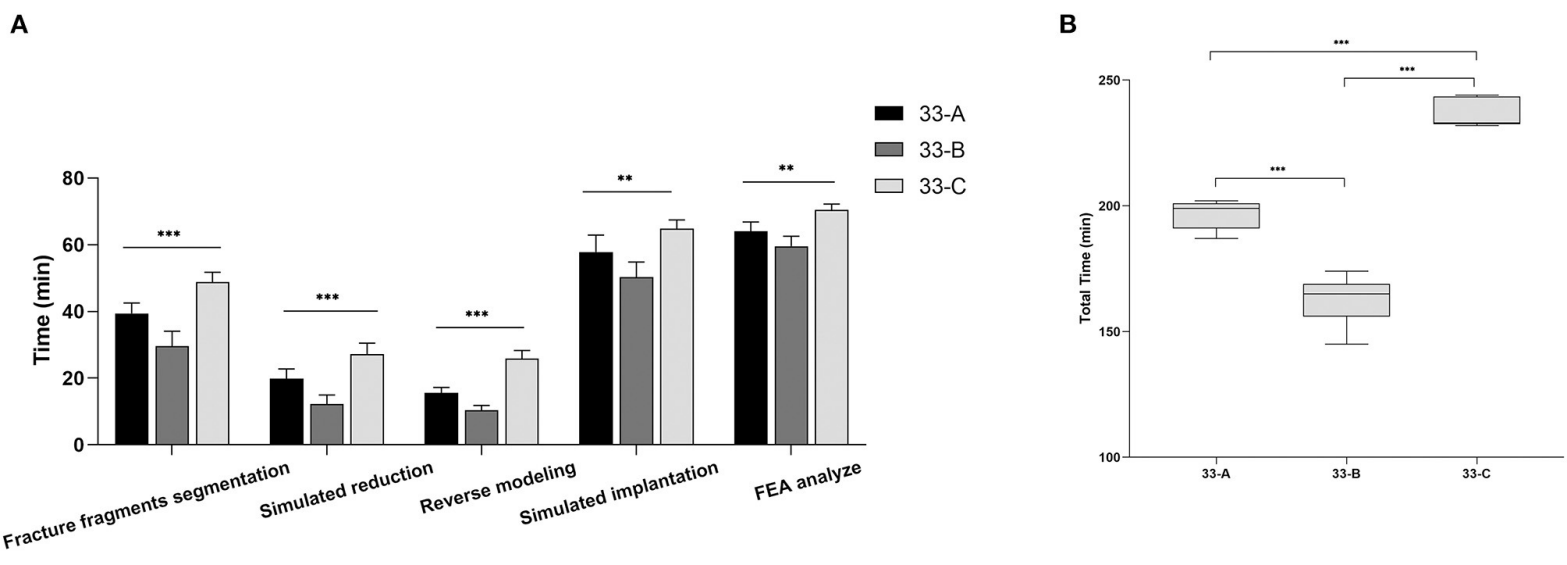
The images show the stages and total time spent in computer-assisted preoperative planning for different subtypes of distal femoral fractures. **(A)** Values are expressed as mean (bars) and SD (error bars). **(B)** The box shows the upper, lower quartile, and the median, the whiskers show the upper and lower limits. **p < 0.01, ***p < 0.001.

The 33-C group took the most time, the 33-A group the medium, and the 33-B group the least times. The difference was significant for all phases (*p* < 0.001). The mean total time required to perform preoperative planning in groups 33-A, 33-B, and 33-C was 194.8 ± 6.49 min (range 184 to 202 min), 163.71 ± 9.22 min (range 145 to 174 min), and 237 ± 5.33 min (range 230 to 244 min). These results suggest that the time required for computer-assisted preoperative planning is significantly correlated with the fracture type. More complex fractures require more time for preoperative planning (Figure 5).

**Figure 6:**
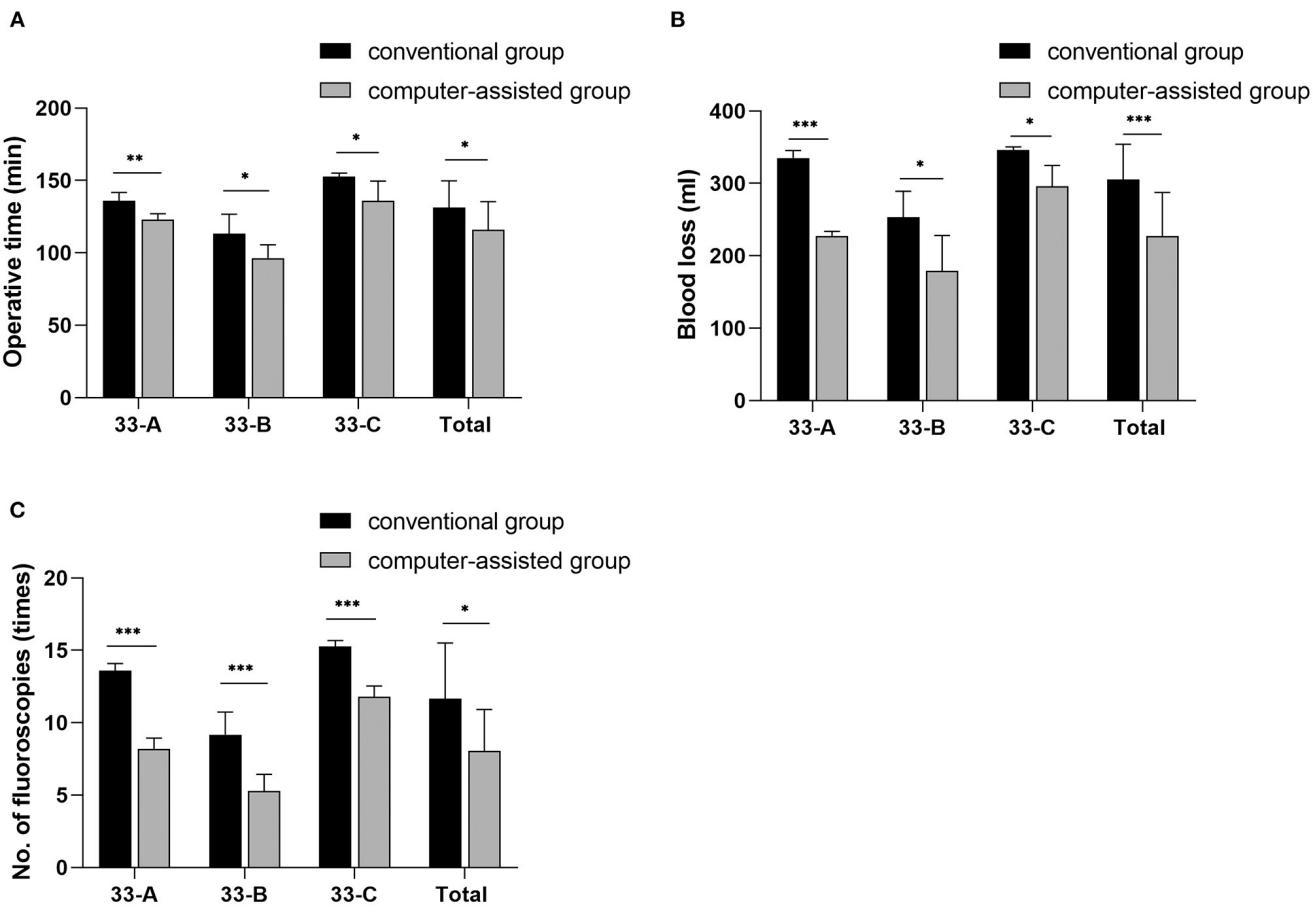
The images show the differences in intraoperative parameters for different subtypes of distal femoral fractures. Values are expressed as mean (bars) and SD (error bars). *p < 0.05, **p < 0.01, ***P < 0.001.

Of the 31 patients, 15 in the conventional group (48.4%) and 16 in the computer-assisted group (51.6%). The mean follow-up was 24.1 months (12 to 36 months) for the conventional group and 25.1 months (12 to 36 months) for the computer-assisted group. In the computer-assisted group, patients had shorter operative time, less blood loss, and fewer fluoroscopic images (Figure 6). This was related to the preoperative surgeon had performed a simulated reduction of the fracture and selected the appropriate plate and screw lengths prior to surgery.

However, although the mean days of hospital stay, mean KSS score, mean VAS score, and mean knee joint range of motion were better in the computer-assisted group than in the conservative group, there were no statistical differences (Table 4). In the conventional group, a 30-year-old diabetic man underwent a second operation for non-healing of a type 33-C fracture. All the fractures in the computer-assisted group healed.

**Table 4:** Clinical outcomes and postoperative rehabilitation.

	**Conventional group (*N* = 15)**	**Virtual surgical group (*N* = 16)**	***P*-Value**
Follow-up time	24.4 ± 6.62	24.24 ± 6.68	0.95
Operative time	131.33 ± 18.48	115.89 ± 19.57	<0.05
No. of fluoroscopies	12.27 ± 2.82	8.06 ± 2.86	<0.05
Blood loss	305.33 ± 48.73	227.65 ± 59.80	<0.05
Duration of hospital stay	12.53 ± 1.89	11.41 ± 1.54	0.08
VAS score	1.73 ± 0.78	1.47 ± 0.98	0.42
Knee society score	159.33 ± 6.43	162.65 ± 6.21	0.08
Range of motion	119.67 ± 9.03	123.82 ± 8.50	0.22

*The values are given as the mean and the standard deviation*.

## Discussion

The distal femur is divided into the supracondylar region and the intercondylar region. The supracondylar region includes the region between the femoral shaft and the femoral condyle, and the intercondylar region includes the region between the femoral condyle and the articular surface (6). Compared to the supracondylar region, the intercondylar region has a richer blood supply and fractures heal more easily. The normal femoral shaft is everted 6 to 11 degrees relative to the joint line. Surgical treatment of distal femoral fractures aims to anatomize the reduction of the joint surface, restore limb length, correct the angle of rotation, and maintain alignment of the fractured ends.

The treatment of distal femoral fractures remains challenging at present as they are often complex, intra-articular, and fragmented. Keeping the soft tissue connected to the bone fragments during reduction may reduce the incidence of osteonecrosis. This requires the surgeon to reduce dissection of the tissue while ensuring an adequate surgical field of view. Restoration of the normal valgus angle and prevention of varus collapse are prerequisites for successful distal femoral reconstruction and good healing. Abnormal angles after reduction increase the incidence of traumatic arthritis and fracture nonunion (7, 18). This requires the surgeon to have a clear understanding of the reduction process before the surgery. In the elderly and patients with osteoporosis, the risk of non-healing of complex fractures is increased due to decreased bone mineral density and decreased trabecular and cortical bone. This requires the surgeon to treat the patients individually during the treatment process to obtain the optimal treatment effect. In addition, in some distal femoral fractures after knee replacement, knee prostheses limit the position of internal fixation, especially screws which play a key role. As a result, surgeons need to spend a long time designing unconventional fixation methods. Various methods have been described in the literature for the treatment of distal femoral fractures, including external fixation, internal fixation, arthroplasty, and so on. In the traditional preoperative planning, the surgeon selects different steel plate and screw combinations according to personal experience and habits, with great uncertainty. The surgeon is not sure if the strength of the selected plate and screw combination is appropriate. A fixation that is too stiff will lead to a slow callous, which may, in part, lead to non-union. A low stiffness allows high deformation and displacements to occur and may lead to the breaking of the plate and screws under heavy load and high strain (19).

These problems can be resolved with good preoperative planning. Several software had been developed for computer-assisted preoperative planning of distal femoral fractures. Wang et al. developed a computer-assisted three-dimensional visualization and operation simulation system based on Unigraphics NX and Mimics. The use of this system was of great significance to help surgeons learn how to treat distal femoral fractures (20). Recently, Chen et al. used a computer-assisted virtual surgery system (Super Image Orthopedics Edition 1.0; Cybermed Ltd, Shanghai, China) to compare the methods and results of conventional preoperative planning and computer-assisted preoperative planning. The software was developed using the Java language on the NetBeans (Sun Microsystems, Inc., Santa Clara, California) and Open Inventor (Mercury Computer Systems / TGS Unit, San Diego, California) platforms. This computer assisted technique has shown satisfactory clinical and radiological results in the treatment of distal femoral fractures (21). However, none of these methods evaluated the appropriateness of the choice of plate and screw combinations in preoperative planning. The choice of internal fixation was still based on a manual technique and surgeon, although it is possible to virtually reset and select different plate and screw positions and lengths. In the face of complex distal femoral fractures, the therapeutic effects of different plate and screw combinations are completely different. Current software cannot help surgeons to select the most appropriate combination of steel plate and screws.

Finite element analysis combined with computer-assisted preoperative planning has great advantages in selecting the appropriate plate and screw combinations. Surgeons can use computer-assisted preoperative planning to design different plate and screw fixation schemes, and then conduct finite element analysis on the biomechanics of the different schemes. By comparing the stress and deformation of different plate-screw combinations, the planning provides theoretical support for clinicians to select the optimal biomechanical combination. As an example in this study both steel plate lengths were considered appropriate for a patient. However, finite element analysis revealed that the force and deformation of the 9-hole steel plate was better than the 8-hole steel plate. Hence, the clinician selected the 9-hole steel plate having biomechanics, and the patient was discharged after rehabilitation (Figure 4).

Moreover, in the process of finite element analysis combined with computer-assisted preoperative planning of different steel plate and screw combinations for different patients, clinicians can have a deeper understanding of the biomechanics caused by different fracture types and the biomechanics of different steel plate and screw combinations. This will help enhance a clinician's understanding of fracture surgery options. The finite element method combined with computer-assisted preoperative planning can also be used for analyzing the bone density of different patients after assigning different material attributes to the bones of the patients, which reflects the individual treatment of the patients. Through the visual results of computer-assisted preoperative planning combined with finite element analysis, surgeons can communicate the advantages and disadvantages of different treatment methods with patients more easily. Compared to 3D printing technology, it is faster and does not need extra cost. Compared with other computer-simulated preoperative planning, the computer-assisted preoperative planning combined with finite element analysis does not require development of new software and the existing software can meet the requirements.

The computer-assisted method used in this study is relatively time-consuming. Advances in fracture fragment segmentation methods will help to shorten this time in the future. Segmenting bones from computed tomography images is a complex task that is affected by the type of fracture. Initially, Tomazevic et al. used different gray thresholds to segment different tissues, but this rough method was not suitable for dividing fracture fragments (22). Paulano et al. used a region growing algorithm, and each CT slice was set with a seeded boundary, which was used to mark each bone (23). Lee et al. used a multi-region growing method based on region growing, which identified the pixel of bone as higher than the threshold intensity of specified seed (24). If the multi-region growth method failed, the fracture fragments were separated by a manual marking method. The fracture fragment division in this experiment was also based on this semi-automatic method. When fracture pieces overlapped, manual intervention was needed and it increasing the analysis time. Buschbaum et al. performed fragment segmentation automatically by reconstructing fracture lines on the surface of fracture fragments. Since fracture lines were used to segment the fracture fragments, this approach did not require any blueprint such as contralateral bone or related medical knowledge. However, for the edge part of the fracture, the algorithm had poor performance (25). Ruikar et al. developed a framework that can automatically assign unique tags to fracture fragments for fragment segmentation through entity removal, segmentation and labeling (26). However, the effectiveness of this method in complex fracture segmentation needs to be further verified. With the further development of machine learning and deep learning, efficient and correct segmentation methods will continue to improve. This will help to reduce the time required for the fragmentation process and support improvements to the computer-assisted preoperative planning software.

There are five limitations to this study. Firstly, when reconstructing the bone model, smaller fracture fragments are discarded to reduce computer operation, but they may play a role in biomechanics. Secondly, some differences were found between preoperative planning and intraoperative implementation. Surgical simulation is performed without soft tissue, allowing implants to be placed in any direction. However, due to the existence of other tissues, the expected position of the implant is not always completely consistent with the preoperative plan. Therefore, the next iteration in this research is to reconstruct the muscles and ligaments, and then carry out virtual placement of implants. Thirdly, this study only included the steel plate fixation, and there were many fixation methods such as intramedullary nail and external fixators used clinically. In the follow up research studies the fixation methods will considered using the finite element method combined with computer-assisted preoperative planning expanding the different fixation methods, to expand the use scope of this method. Fourthly, the software packages used in this research have a steep learning curve related to them. In the follow up research the software packages used will be reduced or the development of a one-stop software package will take place. Finally, the sample size of this experiment is small. The clinical incidence of distal femoral fractures is small, the intention is to collect multi-center cases and analyze the effect of finite element combined with computer-assisted preoperative planning in the follow up research study.

## Conclusion

The results of this study show that finite element combined with computer-assisted preoperative planning can effectively help surgeons to make accurate and clinically relevant preoperative planning for distal femoral fractures, especially in the selection of appropriate plate length and screw positioning.

## Data Availability Statement

The original contributions presented in the study are included in the article/supplementary material, further inquiries can be directed to the corresponding authors.

## Ethics Statement

The studies involving human participants were reviewed and approved by Ethics Committee of the Department of Orthopedics, Beijing Chaoyang Hospital, Capital Medical University (310, 2017). The patients/participants provided their written informed consent to participate in this study.

## Author Contributions

YH: conception and design and data analysis and interpretation. JZ: administrative support. YL and BY: provision of study materials or patients. DW, HW, and PY: collection and assembly of data. All authors: manuscript writing and final approval of manuscript.

## Conflict of Interest

The authors declare that the research was conducted in the absence of any commercial or financial relationships that could be construed as a potential conflict of interest.

## Publisher's Note

All claims expressed in this article are solely those of the authors and do not necessarily represent those of their affiliated organizations, or those of the publisher, the editors and the reviewers. Any product that may be evaluated in this article, or claim that may be made by its manufacturer, is not guaranteed or endorsed by the publisher.
